# Health research priority setting in Zambia: a stock taking of approaches conducted from 1998 to 2015

**DOI:** 10.1186/s12961-016-0142-z

**Published:** 2016-09-23

**Authors:** Pascalina Chanda-Kapata, William Ngosa, Busiku Hamainza, Lydia Kapiriri

**Affiliations:** 1 Department of Diseases Surveillance Control and Research, Ministry of Health, Lusaka, Zambia; 2 National Malaria Control Centre, Ministry of Health, Lusaka, Zambia; 3 Department of Health, Aging and Society, McMaster University, Hamilton, ON L8S 4L8 Canada

**Keywords:** Priority setting, Zambia, National health research system, Research for health

## Abstract

**Background:**

Priority setting in health research is an emerging field. In Zambia, like many other African countries, various priority setting activities have been undertaken with a view to identify research activities to which the available resources can be targeted while at the same time maximising the health impact for resource allocation to support evidence-based decision-making. The aim of this paper is to document the key elements of the various priority setting activities that have been conducted since 1998, identifying the key lessons and providing recommendations to improve the process.

**Methods:**

A comprehensive review of the previous priority setting activities and processes in Zambia was conducted. Both published and unpublished reports were reviewed in order to identify any research priority setting processes that have been undertaken in Zambia. We developed a framework, based on the priority setting literature, to guide our abstraction and synthesis of the literature.

**Result:**

The earliest record of priority setting was conducted in 1998. Various priority setting approaches have been implemented in Zambia; ranging from externally driven, once-off activities to locally (in country) initiated comprehensive processes. However, there has been no systematic national process for priority setting. These priority setting processes in Zambia were characterised by limited stakeholder buy-in of the resulting national research or programmatic research agenda. Most striking was the lack of linkages between the different initiatives. There seems to have been no conscious recognition and building on previous priority-setting experiences of previous initiatives.

**Conclusion:**

There were gaps in the priority setting processes, stakeholder engagement and application of a defined criterion. There is a need for a priority setting framework coupled with local capacity developed across a range of stakeholders.

## Background

Prioritisation of health research is an area that requires attention in the light of scarce resources considering its importance in ensuring that the relevant evidence generated from it addresses national priorities [1]. Since the provision of health services has become entirely dependent on quality and timely availability of evidence-based decision-making, research has assumed a strategic and important role in providing new scientific knowledge and insights. The research priorities tend to determine the research agenda, practices and technologies of a given national health research system [2]. Besides voicing of research priorities and strategies, the maximisation and utilisation of research outcomes are extremely important ingredients in this process.

Most low- and middle-income countries face severe resource constraints, making it difficult for sufficient resources to be allocated to the health sector and health research. Priority setting is important because it guides investments in healthcare, health research and respects resource constraints [3]. Setting priorities for health research is essential to maximise utilisation of the meagre resources allocated to the health sector and is regarded as a key factor in the effort to strengthen national health research systems [4], especially in low-income countries, where government expenditures on health are less than US$ 20 per capita per year [5].

A 1990 report by the Commission on Health Research for Development created momentum for researchers and policymakers to become interested in priority setting both at country and international levels [6]. It recommended that countries should develop a national plan for conducting health research and that each country should set its own priorities for health research. As a result, a number of low- and middle-income countries, such as Zambia, began to experiment with setting priorities for health research to guide various stakeholders from the health and non-health sectors [7].

Zambia’s health research system has undergone a great deal of growth. Until recently, there was no single governance body that provided leadership in health research. The responsibility was shared between the Ministry of Health and Ministry of Science, Technology and Vocational Training. A major breakthrough was scored when the National Health Research Policy was approved in 2010. This policy provided strategic direction for the promotion, conduct, prioritisation, financing and institutionalisation of health research [8]. This culminated into the enactment of a landmark piece of legislation in March 2013. The Health Research Act, among other things, provided for an institutional framework for the prioritisation of areas for health research, dissemination, monitoring and evaluation, and a Trust Fund for funding national research priorities [9]. The identification and prioritisation of areas for research and eventual funding would help the country not to rely on external donors when deciding which areas of research to fund as donor priorities may not always conform to national priorities [10].

A significant amount of health-related research has been carried out in Zambia over the past decade. However, while the process of identifying research gaps has been ongoing since 1998, there has been no sustainable system for regularly coordinating research priorities. Priority setting for health research has been ad-hoc, with little consideration for ongoing or previous health research activities [11]. Additionally, there has been no systematic synthesis of the approaches and lessons learnt from previous priority setting activities. A synthesis of these lessons would be critical in informing future efforts. We present here a description of some of the priority setting approaches which have been conducted in the country from 1998 to date.

## Methods

A comprehensive review of the previous priority setting activities and processes in Zambia was conducted. Both published (peer reviewed) and unpublished reports (institutional documents) were reviewed in order to identify any priority setting processes which have been undertaken in Zambia. The search terms for the published literature included: “Zambia”, “priority setting” and “health research”. The databases searched included the COHRED Website and PubMed. Five retrieved articles were relevant for this analysis, of which four were reports from priority-setting exercises undertaken by the Ministry of Health (MoH).

The literature review was undertaken between September 2014 and June 2015. The identified records were reviewed in order to identify any priority setting processes involving health research regardless of the focus of the priority setting.

We developed a framework, based on the priority setting literature [12], to guide our abstraction and synthesis of the literature. This framework included aspects that are deemed relevant to priority setting, e.g. explicit processes used, stakeholder involvement, guiding framework/approaches employed, criteria used, and the outcome of priorities once set. The abstracted information was synthesised and summarised according to these themes. In the results section, we present each priority setting initiative and discuss the details under each of the identified themes.

This study received ethics clearance from McMaster University and The University of Zambia Humanities and Social Science Ethics Committee, IRB # 00006464. Permission to conduct the study was received from the MoH in line with local guidelines.

## Results

Our review revealed that, to date, five health research priority setting initiatives have been undertaken in Zambia, namely (1) the National Health Research Advisory Committee (NHRAC) of the MoH initiative; (2) priority setting for health research as part of the general priority setting for health driven by the National Health Strategic Plan 2006–2011; (3) priority setting by the National Science and Technology Council (NSTC); (4) priority setting by The Zambia Forum for Health Research (ZAMFOHR), a comprehensive priority setting process for MoH programs; and (5) priority setting by the MoH in partnership with the World Health Organization Implementation Research Leverage Fund (WHO-IRLF) on Maternal, Neonatal and Child Health (MNCH); we discuss each in detail (Table 1). Overall, the priority setting processes which have so far taken place fall short of the fairness criteria described by Kapiriri et al. [13] since information on publicity, revisions and enforcement was found to be lacking.

**Table 1:** Summary of priority setting process, success, challenges and recommendations

Title	Process	Success	List of identified priority areas	Challenges	Recommendations
Ministry of Health (MoH) 2008, Country Report Alignment and Harmonization in Health Research	− Tracking what research had been done − Small group brainstorming sessions − A National Health Research meeting that brought together different stakeholders (200 to 300 people involved) − Synthesis of key research findings by a team of experts	− Integration of the various processes into a coordinated system − Development of processes through which research outcomes could be continually fed into policymaking and programme implementation − Identification of a process for updating the priorities	− Malaria − Child health − Nutrition − Diarrhoeal diseases − Reproductive health − Sexually transmitted diseases/HIV/AIDS/tuberculosis/leprosy − Water and sanitation	− The process was disease focused, which affects priority research − Process was not a very representative or inclusive − The influence of donors was also cited as a problem that skews health research priorities − Current donor interest in certain conditions influences the availability of research funds	− Formalised process be established for participatory health research − Priority setting and review with a clear listing of all relevant stakeholders to be involved
August 2010 to March 2011, Zambia Forum for Health Research	− Worksheets for summarising the approach for prioritising topics for policy briefs were given to participants − The participants were then divided into working groups − The scope of the priority-setting process was limited to the topic of sexual and reproductive health	− All stakeholder groups (those that would be affected by the outcome of the policy) were represented at the workshop and all participated in the process; this enabled a wide range of reproductive health priority topics to be identified − The method used was transparent − Participants had an opportunity to take an active part in compiling the list of priority topics	− Promoting the use of misoprostol in labour specifically to prevent haemorrhage after delivery at home − Ensuring that all maternal deaths are notifiable − Encouraging research at the district level − Fostering the involvement of traditional leaders in reproductive health programmes	− The input of some participants was overshadowed by those who were more outspoken; some vital contributions may have been missed as a result − Some important topics, including the reasons for the drastic decline in facility-based births; tackling abuse at facility-based births emerged only after the workshop; these topics were provided by individuals who were unable to attend the workshop	− Adopt a standard national priority-setting tool − Financing of priorities for research
2011, MoH Program Managers	Using the Medium-Term Expenditure Framework approach	- Had a comprehensive list of priority setting lists for the all country	− Child health − Cancer diseases hospital − Reproductive health − Health education − Oral health − Pharmacy − Non-communicable diseases − Virology laboratory − Malaria − Antiretroviral therapy − Nutrition − Tuberculosis − Mental health and substance abuse	− Period to get consensus from all stakeholders was short − Not all stakeholders were involved in the process, especially the community − Implementation was affected by lack of resources to carry out the priority setting research	− Adopt a standard national priority-setting tool − Allocate resources to implement the priority areas identified − Enforce the National Health Research Act No 2 of 2013
October 2011, Maternal, Neonatal and Child Health Priority Setting Case, Zambia	Child Health and Nutrition Research Initiative method	− It was short, focused − It was responding to a specific program area − It was easy to identify stakeholders − It was transparent	− How can community-based neonatal care be strengthened to reduce mortality and morbidity in Zambia? − How can strategies to reduce adolescent/teenage pregnancies be strengthened in Zambia? − How can we improve the proportion of institutional deliveries? − How can child immunisation coverage be improved? − What is the effectiveness of different models to attract and retain doctors, nurses, and technicians in rural and hard to reach areas? − What incentives can be used to improve attendance for postnatal care in Zambia? − How can we improve early ANC attendance in the first trimester?	− It was externally driven − The was no post evaluation of the process or follow-up	− Priority-setting activities should be locally driven and standardised − Global partner should buy into national priorities as is being recommended by WHO ESSENCE
2007 to date (Annually) National Science and Technology Council Strategic Research Fund	Receiving priority areas from all sectors	− Multi-sectoral and funds are allocated to fund research − It is transparent − There is standard call for proposals − Standard proposal evaluation − The funding is predictable − It is efficient and locally driven	− Communicable diseases − Non-communicable diseases − Maternal diseases − Nutritional diseases	− Process for selecting the priority areas in each sector is not standardised though guided by the Five-Year National Strategic Development Plans	Standardised intra-sector priority setting

The first priority-setting exercise was conducted in 1998 by the NHRAC of the MoH. The Ministry of Science, Technology and Vocational Training was involved through the NSTC, which is a statutory body that oversees research for all sectors in the country. The MoH was involved through the NHRAC, which was established in 1998 to monitor developments and identify needs for action in health research [14]. This exercise involved tracking what research had been done through small group brainstorming sessions. A National Health Research meeting was called, bringing together different stakeholders. The stakeholders were composed of representatives from government program officers, researchers, funding agencies, non-governmental organisations (NGOs), and academia. The details of the nature of the stakeholders were not clearly stipulated, but an estimated 200 to 300 people were part of the process. After this, a team of experts synthesised the input from stakeholders. Thereafter, a National Health Research meeting was held, bringing along different stakeholders to further synthesise key research findings. This resulted in a list of recommendations which were then submitted to a sub-committee of experts to analyse, refine and package the extensive list that came out of the meeting. The committees’ effort was subjected to a consensus meeting that provided additional feedback and input. The seven national health research priority areas which were obtained from this exercise were malaria, child health, nutrition, diarrheal diseases, reproductive health, sexually transmitted disease (including HIV/AIDS), tuberculosis, and water and sanitation. There are no reports to show the further break down of these priority areas. The priority areas were then disseminated in 1999 though hard copies and emails. After the priorities were set, funding agencies were informed to help mobilise resources to fund the identified priorities. However, there was no evaluation of the extent to which these priorities were implemented [14].

### The lessons learnt

The main positive outcome was that this initiative integrated the various priority setting processes into a coordinated system, thereby developing processes through which research outcomes could be continually fed into national level policymaking, programme implementation, and identification of a process for updating the priorities. However, there were several challenges associated with this initiative. First, there were limited funds allocated to conduct research in the priority areas because the MoH continued to focus on curative services. Second, although provincial and district officers, NGOs, members of parliament and researchers were represented, the process was perceived as not being representative and inclusive by certain sections of the research community [14]. Third, according to some researchers, donor interests contributed to skewing health research priorities, whereby funds were only available for the research issues donors were interested in. As a result, limited funding was only available for tuberculosis, malaria and HIV.

For the period 2006 to 2011, the priority setting for health research was part of the general priority setting for health driven by the National Health Strategic Plan 2006–2011 developed by the MoH [15]; although it primarily focused on the health sector, it also fed into the Fifth National Development Plan for Zambia, which is part of the Medium-Term Expenditure Framework [16]. The annual planning cycle started with the Provincial Medical Offices and other provincial officials attending the national planning launch at the MoH’s national offices, where guidelines and central issues for consideration in the following year’s budgets are presented and discussed. The Provincial Medical Offices were presented with indicative figures from the central level (MoH) which each district within the province is expected to follow when setting their priorities [16]. The main lesson learnt here is that priority setting for health research may also take place as part of the general priority setting for health.

The NSTC has been conducting annual priority setting since 2007. It is not very clear what framework they use, but the process involves receiving priority areas from all sectors (namely agriculture, health, education, energy, commerce, mines, defence, etc.). The criteria for coming up with the sector-specific priorities is not predetermined, but is largely informed by the sector strategic plans and the national development plans. The priorities submitted from each sector are reviewed by the Sector Advisory Group, composed of sector representatives with a background in research and development, who select the final list of priority areas. Once the Sector Advisory Group approves the final list of research priorities, it constitutes the list used for a call for proposals. The call is advertised using the national print media and online. Since it is an annual process, the priorities are revised and updated based on monitoring findings of the previous year’s performance.

### The lessons learnt

The process is multi-sectoral and funds are allocated to fund the identified priority research areas. It is transparent, with a standard call for proposals, standard proposal evaluation and predictable funding. Furthermore, it is efficient and most important locally driven. The disadvantage with this process is that, although guided by the Five-Year National Strategic Development Plans; the priority processes within each sector are not standardised. Furthermore, the total number of projects which have been funded through this mechanism could not be verified.

ZAMFOHR, an NGO, also conducted a priority-setting exercise for sexual and reproductive health between August 2010 and March 2011 on behalf of the MoH. Participants ranged from public servants, community-based organisations and researchers working on sexual and reproductive health. The process involved giving summarised worksheets of topics to participants. The initial list of priorities was developed by the Technical Committee on reproductive health. The participants were then divided into working groups and generated research topics. The topics which were generated were ranked by the two groups; the details of the process are summarised in a report [17]. Information on further publication and funding of priorities was not available.

The process was participatory involving stakeholders working in reproductive related programs. The method used was transparent and participants were actively involved in compiling the list of priority topics. Input from stakeholders who were not present at the workshop was received after the workshop. However, some participants who were present may have been overshadowed by their outspoken counterparts, leading to some vital contributions being left out [17].

A comprehensive priority-setting process for MoH programs was performed in 2010, whose purpose was to provide the government and partners with priority health research areas for resource allocation, mobilisation and implementation. The process was initiated by the Research Unit of the MoH creating guidelines or rules of engagement for the process. These guidelines were disseminated to program managers within the MoH. After that, each program manager convened a technical working group (TWG) in their respective areas of service delivery. These included malaria, child health, reproductive health, non-communicable diseases, mental health, HIV/AIDS, tuberculosis and health system strengthening. The program-specific TWGs deliberated on issues affecting the implementation of their respective programs. After deliberation, a list of potential study areas was identified. The list was submitted to the secretariat for compilation. The secretariat did not further synthesise the lists provided. After all TWGs had completed their consultations, a national dissemination meeting comprising funders, academicians, implementers and policymakers was held; the meeting involved about 100–150 participants. The compiled priority areas were presented to the stakeholders for further discussion in a one-day workshop held at national level. The discussion was moderated by the national research TWG of the MoH. All the meeting participants then provided further input either by adding more priorities or refining the phrasing of the priorities; others who needed extra time for submission were requested to do so through email. Finally, a final list of research priority areas was developed in form of a document and submitted to stakeholders for reference [18]. As a result of this exercise, some priority research areas in malaria, tuberculosis, maternal health and health system strengthening were funded and conducted during the period under review [19]. The other areas which were not funded were still maintained on the priority list; this is what was termed as a research agenda. As an example of partners using the outcome of this priority-setting process, the MoH, in collaboration with 3DE program (an initiative of the Clinton Health Access Initiative and IDinsight in partnership and funded by the Department for International Development) began conducting selected impact evaluations [20–23]. The projects were selected from the national priority areas for research [18]. The main lesson learnt from this general priority-setting process was that a national research system with already set priorities is likely to attract stakeholder buy in and may lead to implementation of some of the set priorities. Additionally, policy relevant research is conducted, and thus the likelihood that such research will inform local policies is high [24].

The MoH, in partnership with the WHO-IRLF on MNCH, conducted a priority-setting process for implementation research in October 2011. The participants were drawn from local research institutions and provincial/district level MNCH implementers (academia, NGOs, private and public). Participants received an introductory presentation on implementation research. After that, the Child Health and Nutrition Research Initiative (CHNRI) method [25, 26] for priority setting was introduced to the participants as a tool to apply during the priority-setting process. Thereafter, participants were split into small working groups to brainstorm on potential implementation research questions which could be implemented in order to improve the MNCH situation in the country. Each group compiled a ‘shopping list’ of questions. After group presentations, all the questions which were identified were pooled into a long list of research questions. The participants were then requested to apply an objective ranking method by attaching scores to come up with a form of ranking using the CHNRI method. The criteria of selection included feasibility of the research being conducted, cost, likelihood of informing policy, and nature of the question. The results of the priority setting were transparently displayed in order of the questions getting the highest score. The top scoring 10 research questions were then adopted as the priority areas for MNCH implementation research in Zambia [27]. The list was communicated to all stakeholders working in MNCH in Zambia. There was no provision for the amendment of the list after the workshop. One proposal was funded; however, there was neither post-monitoring/evaluation of the process nor documentation of the impact of the study conducted. The main lesson learnt from this process was that, when setting priorities, there is an assumption that there exists adequate capacity to conduct research on the identified priorities. In this case, it was observed that the researchers did not apply themselves to the requirements of implementation research. This led to low implementation of the set priorities even though the funding to implement the research activities was available from the funding agency that conducted the priority setting.

## Discussion

Various priority-setting approaches have been implemented in Zambia; ranging from externally driven, once-off activities to locally initiated comprehensive processes. It was found that there was a lack of linkages between the different initiatives, there seems to have been no conscious recognition and building on previous priority-setting experiences between these initiatives. Each seems to have been a stand-alone, and often one-off, initiative. This paper fills this gap by providing a synthesis of these initiatives, identifying key lessons which can inform future priority-setting initiatives.

Our analysis showed that all but one of the priority-setting activities had some explicit process of participatory nature. However, the general public was not involved in any of the priority-setting activities. General stakeholder involvement was evidently being practiced. There was a lack of a clearly defined priority setting framework guide applied, with the exception of the MNCH, which used the CHNRI method. Additionally, all but one did not have explicit priority-setting criteria and there was a general lack of implementation and monitoring plans. With the exception of the MNCH CHNRI method, there was no incentive for adhering to the set priorities.

The availability of a clear policy environment and, most recently, a legal framework, promises to help improve the manner in which priority setting is conducted. There will be a need to develop priority-setting expertise locally so that the process can be sustained. In order for this to happen, it is proposed that a priority setting institute be developed in country with the goal of building local capacity.

The common issue for all the activities which were undertaken was the obvious involvement of technical experts and technical committee members (funders, implementers, academicians, etc.). However, the major disadvantage was the exclusion of the community level participants. Nonetheless, the gate keepers of community interests were all involved, so it can be argued that there was some representation or attempt to reflect the needs of the communities. The presence of district stakeholders in a priority-setting meeting has been shown to have potential to reflect community interests for health intervention priority setting in Kenya, Tanzania and Zambia [28]. It is yet to be established whether this is true for health research priority setting.

The priority-setting exercises which were locally driven, such as the one by the Ministry of Science and Technology, appeared to have led to research activities being implemented. This underscores the need for priority setting to be institutionalised within the local health system and increases the chances of success in terms of funding allocation, participation of stakeholders and likelihood of yielding actual research activities. This is an important lesson if priority setting is to be viewed as an important function of the health research system in the country.

Another observation was the limited capacity among stakeholders to apply themselves appropriately to the set priority areas. Therefore, apart from capacity building, incentive mechanisms for adhering to the set priorities need to be developed. Enforcement of the legal framework will be cardinal to ensure that the results of the priority-setting exercise are adhered to. One of the causes for low adherence was also the fact that funding for research is largely external [29]. The fact that the funding for health research mostly comes from external sources has implications for the identification and setting of priorities for health research. In order to minimise external influence on research priorities, the legal framework stipulates that only research identified on the priority list will be funded using government resources [8]. Other modalities include holding consultative meetings with funding agencies to include national work plans in their funding calls. A good example is the European & Developing Countries Clinical Trials Partnership funding modality, where countries provide input into what they would like to see in international calls for funding; the sources of information are the country work plans submitted prior to calls being made [30].

The process of allocating funds for research provides only limited incentives for researchers to focus their work on identified priorities. A standard national priority setting tool is also required to ensure uniformity of approaches. Such a framework should be based on the lessons learnt to date from the Zambian experiences with priority setting for health research and should include (1) an explicit framework, that is chosen or agreed upon by the relevant stakeholders to guide the process; (2) clear mechanisms for monitoring and a framework for evaluation of the priority setting process and the implementation of the identified priorities; (3) annual feedback mechanisms to ensure the implementation of the identified key lessons; and (4) a clear priority-setting process, explicit criteria and processes for ranking the research options, clear mechanisms for wide stakeholder engagement – especially those from the sub- national levels, and mechanisms for considering a wide variety of evidence to guide priority setting.

There is need for strong global forums where country experiences can be shared. This will further serve to not only validate available frameworks but also provide ongoing processes for improving priority setting. The global forums can also serve as opportunities to engage international stakeholders on the importance of adhering to national health research priorities. One such example is ESSENCE on health research, which is an initiative of funding agencies to improve the coordination and harmonisation of research capacity investments [31] and to harmonise their activities and procedures with the priorities of the countries in which they operate ﻿[﻿^32^] according to the principles of the 2005 Paris Declaration on Aid Effectiveness and the 2008 Accra Agenda for Action [33]. 

Future research on how the priority-setting processes used and challenges faced by Zambia are relevant to other countries facing similar tasks is recommended.

## Conclusion

This paper presents the first synthesis of lessons learnt from the health research priority setting initiatives in Zambia. We found that locally driven processes appeared to have yielded more positive results than externally driven processes. There appears to be systems within which priority setting can be institutionalised in the health system of Zambia both in the health sector and at a multi-sectoral level. Enforcement of the legal framework and implementation of incentives for promoting adherence to national research priorities will be necessary if the health research system is to benefit fully from a well organised priority setting process. However, the lack of strong monitoring mechanisms makes evaluation difficult. There is a need for standardised priority setting approaches with well thought through monitoring mechanisms so that the process can be improved. Non-representation and inadequate funding of identified priorities still remains a challenge.

## Abbreviations

CHAI: Clinton Health Access Initiative
CHNRI: Child Health and Nutrition Research Initiative
IRLF: Implementation Research Leverage Fund
MNCH: Maternal, Neonatal and Child Health
MoH: Ministry of Health
NGOs: non-governmental organisations
NHRAC: National Health Research Advisory Committee
NSTC: National Science and Technology Council
TWG: Technical Working Group
ZAMFOHR: Zambia Forum for Health Research
